# Management and Best Means of Preventing the Destruction of the Deciduous Teeth

**Published:** 1867-10

**Authors:** Frank French


### MANAGEMENT AND BEST MEANS OF PREVENTING THE DESTRUCTION OF THE DECIDUOUS TEETH.

BY DR. FRANK FRENCH.

Read before the Western New York Dental Society.

In treating this subject I wish, in the first place, to go directly at the root of the matter, viz.: the formation of these teeth. At as early a period as the sixth week after conception, the preparation of a receptacle for the deciduous teeth can be discerned in the fœtus, consisting of a deep groove extending around each jaw, lined with mucous membrane, the posterior part of which is divided by a small ridge into two grooves, and as the germs of the teeth appear in the inner one, it is commonly called the primitive dental groove. By the seventh week a small substance in shape like a grain of wheat lying on its side, makes its appearance in the dental groove in the upper jaw, rising up from the mucous membrane, and is the germ of the first temporary molar. In the eighth week the germs of the temporary cuspidati make their appearance in a rounded granular form. The germs of the incisors show themselves during the tenth week, the central first, and soon afterward the lateral, in the form of mucous papillæ. The sides of the groove in the region of the temporary molar are now approaching each other, and during the tenth week processes are sent off from its sides, before and behind it, inclosing it in a follicle or cavity. At the same time the cuspidate is being likewise inclosed. Near the close of the tenth week, the germ of the second temporary molar shows itself. Up to this time the germs of the incisors have advanced slowly, but now begin to grow rapidly, and during the eleventh and twelfth week they are also each inclosed in a separate follicle in the same manner as the others. The second molar is also increasing, and during the thirteenth week is also inclosed. All the germs now begin to take shape, the incisors like the future teeth, cuspidate, form of cones, etc.: during the fourteenth week the lips of the primitive dental groove meet together almost like valves, so that the germs of the teeth are almost hidden. It is about this time that provision is made for the production of ten anterior permanent teeth, and consists of crescent shaped depressions immediately behind the inner covering of the follicles or cavities which inclose the germs of the temporary teeth; first the centrals, then laterals, cuspids and first bicuspids. The dental groove is now soon closed by the adhesion of its lips and walls, and changes its follicles into sacs, the germs into the pulps of the temporary teeth, and the crescent shaped depressions into cavities of reserve. from which the sacs and pulps of the permanent teeth are developed. At the fifth month the germs of the permanent teeth first appear, and at the sixth month bony septa are thrown across the alveolar groove, and little cavities are now formed on the posterior walls of the alveoli for the sacs of the permanent teeth. It is not necessary to follow on through dentification, formation of enamel and the various stages until the tooth is erupted; and my only object in referring to it at all is, to show at what period of intra-uterine existence the formation of the teeth begin, as I wish to show that at least one of the means of preventing the destruction of the deciduous teeth may be used at as early a period as the teeth begin to form. Let us look for a moment at a tooth, and see what it is composed of. Take the dentine: First, and we find phos. lime 62, fluate 2, carb. 5.5, phos. magnesia 1, soda and muriate soda 1.5 gelatine and water 28. This is from dentine as found in the mouth. When dried it varies slightly from this. In the enamel we find phos. lime 85.3, fluate 3.2, carb. 8, phos. magnesia 1.5, soda and nitrat soda 1, animal matter and water 1. Thus in the dentine we find 72 per cent. of inorganic matter, in the enamel

99 per cent., and in the cementum 7 per cent.; so that in the structure of the tooth there is 83 per cent. of inorganic matter, over two-thirds of which is phosphate of lime. In order to have a healthy solid tooth structure, capable of resisting the action of external agents, it is necessary that the system should be well supplied with the materials for construction; and nature has provided that it should be received through the medium of the food. We do not ask or expect a man to build a large solid building with nothing but sand and lime; but it would be just as reasonable as it is to expect nature to perform her work well with the materials that are furnished her. What is usually the food of a woman during the period of gestation? As soon as she knows she is pregnant she begins to be notional, or thinks she must be; her appetite is fastidious and must be tempted, and the whole round of cookery is brought into requisition to satisfy it. Sponge cake, pound cake, cream cake, fruit cake, of which a good sized cake would be almost certain death to the best ostrich that was ever made, and an endless variety of what are usually termed nice things, all made very rich and from the very finest of flour, which is well known contains hardly any of the phosphates. I think that a large proportion of the food of the females of the better class during this period is of articles made from fine flour. Tea is also usually drank in large quantities, and sometimes beer and wine, neither doing any good and productive of harm to the child. Again, she has a morbid appetite, and yearns, or as it is usually termed, longs for things which never ought to be eaten; and because she longs for them must have them, or the child would be marked with the article for which she longed. I knew of a lady in this condition who longed constantly for cauliflower, subsisting almost entirely on this and bread and tea for weeks, her husband scouring the town from one end to the other to procure it, until the poor man became almost certain that the child would be born with a cauliflower in each hand, or at the best would be a cabbage head. The larger portion of the food taken at such times does not contain the material to produce good healthy bone and muscle, and the wonder is not that nature does so little, but that she does so well. There is another point which I wish to notice (although I expect some will take exceptions to it, I am satisfied there is considerable truth in it), and that is the condition of the parents when conception takes place. In many cases it occurs at a time when one or the other, and perhaps both, are in poor health, or are suffering from excessive mental or bodily fatigue, and as a consequence are not only peevish and irritable, but the whole system is deranged, and not able to perform its functions in a healthy manner; and conception taking place under such circumstances must be more or less affected by it. Again, it takes place when there is an unwillingness on the part of the mother to bear children, who, as soon as she discovers that conception has taken place, makes every effort to rid herself of it, and failing in this (the effect of which is often seen in the child) she tries to forget it by a round of fashionable life and dissipation not only endangering her own health, but entailing misery upon her offspring for their whole lives, so that from the period of conception till birth she considers it a curse, and the child is literally born with the mother's curse upon it; and thousands of such cases can be pointed out where from this very thing the child not only suffers physically, but often mentally; the curse clings to it through life. Can it be expected that nature can do her part well with no effort made to aid her, but everything against her? We treat our animals in a more rational manner than we do ourselves. Do we allow them to come together for intercourse when sick, emaciated or worn out with hard work, or over heated from exercise? By no means; it is never thought of; the utmost care is taken to have them in a fine healthy condition, and after conception has taken place they are well looked after to see that they have proper care, are properly kept and have the right kind of food. Now why do we exercise all this care? Because we know that it is necessary for the well-being of their offspring. We want a proper development of bone and muscle and tissue, without which they would be worthless, and to secure which we are willing to take all this trouble. Now what I have to say is all summed up in this: when we are willing to take the same care of ourselves that we do of our animals, when we are willing to devote the same amount of time and study and care, to perfect the development of our own species, that we willingly give to improve the breed of our horses, when we recognize the fact that sexual intercourse was not designed by our Creator as a mere gratification of the baser animal passions, but was intended as a means for the development of the human species, when men and women desire perfect and healthy offspring, and to secure which they are willing to put themselves in proper condition before coming together to beget such offspring, and when once begotten to take proper care of the body and to partake of such food as is not only the most nourishing, but contains at the same time the requisite quantities of such matter as nature requires for the proper development of bone and muscle, and to follow them up carefully not only through the whole period of gestation, but of lactation, and also of early childhood, when we are willing to act upon this plan, then, and not till then, shall we have a perfect development of bone and muscle, such a one as will give a solid healthy tooth structure capable of resisting the attacks of external agencies, that will not only produce deciduous teeth that will last their allotted time, but will also produce solid permanent ones that with care and watchfulness will perform through life the part they were originally designed to. This I consider the best means of preventing the destruction of the deciduous teeth.

